# Metformin in autosomal dominant polycystic kidney disease: experimental hypothesis or clinical fact?

**DOI:** 10.1186/s12882-018-1090-3

**Published:** 2018-10-22

**Authors:** Antonio Pisani, Eleonora Riccio, Dario Bruzzese, Massimo Sabbatini

**Affiliations:** 1 Department of Public Health, Chair of Nephrology, University Federico II of Naples, Via Pansini 5, 80131 Naples, Italy; 2 Department of Public Health, Chair of Statistics, University Federico II of Naples, Naples, Italy

**Keywords:** Autosomal dominant polycystic kidney disease, Metformin, Chronic renal failure

## Abstract

**Background:**

Autosomal dominant polycystic kidney disease (ADPKD) accounts for 8–10% of end-stage chronic kidney disease (CKD) patients worldwide. In the last decade, the advanced knowledge in genetics and molecular pathobiology of ADPKD focused some aberrant molecular pathways involved in the pathogenesis of the disease leading to controlled clinical trials aimed to delay its progression with the use of mTOR inhibitors, somatostatin or tolvaptan. Preclinical studies suggests an effective role of metformin in ADPKD treatment by activating AMPK sensor. Clinical trials are currently recruiting participants to test the metformin use in ADPKD patients.

**Methods:**

We retrospectively examined the records of our ADPKD patients, selecting 7 diabetic ADPKD patients under metformin treatment and 7 matched non-diabetic ADPKD controls, to test the effect of metformin on renal progression during a 3 year follow-up.

**Results:**

During the first year, the GFR decreased by 2.5% in Metformin Group and by 16% in Controls; thereafter, renal function remained stable in Metformin Group and further decreased in Controls, reaching a 50% difference after 3 years of observation. Accordingly, the overall crude loss of GFR, estimated by a linear mixed model, resulted slower in the Metformin than in Control Group (− 0.9; 95% C.I.: -2.7 to 0.9 vs - 5.0; 95% C.I.: -6.8 to − 3.2 mL/min/1.73 m2 per year, *p* = 0.002).

**Conclusions:**

Our data are suggestive of a beneficial effect of metformin on progression of ADPKD. Large, randomized, prospective trials are needed to confirm this hypothesis.

## Background

Autosomal dominant polycystic kidney disease (ADPKD) accounts for 8–10% of end-stage chronic kidney disease (CKD) patients worldwide [1]. In the last decade, the advanced knowledge in genetics and molecular pathobiology of ADPKD focused some aberrant molecular pathways involved in the pathogenesis of the disease [2, 3] leading to controlled clinical trials aimed to delay its progression with the use of mTOR inhibitors [4], somatostatin [5], or tolvaptan [6] which, to date, is the only approved drug for ADPKD treatment. Thus, new experimental and clinical research is still ongoing.

Recently, preclinical studies have suggested that metformin, worldwide used in type 2 diabetes, could play some role in treatment of ADPKD by activating the metabolic sensor AMP-activated protein kinase (AMPK) [7, 8]. Activated AMPK inhibits the cystic fibrosis trans-membrane conductance regulator (CFTR), which suppresses the secretion of fluid and electrolytes into renal cysts, a critical process for their expansion [9, 10]. Moreover, AMPK also phosphorylates tuberin, an indirect inhibitor of the mTOR pathway [11, 12], which regulates tubular cell turnover and whose abnormal activation leads to proliferation of tubular cystic cells and to apoptosis of normal tubular cells. Therefore, AMPK hinders two important pathways involved in ADPKD progression: this strongly suggests that its activation by metformin could represent a therapeutic tool in renal cystic diseases. Accordingly, new clinical controlled trials are currently recruiting participants to test the safety, tolerability, and efficacy of metformin in ADPKD patients; unfortunately, their results will not be available for the next years.

Since ADPKD patients may also suffer from type 2 diabetes [13], we have retrospectively examined the records of all ADPKD patients in regular follow-up at our CKD Clinic between January 2012 and March 2017, to select diabetic ADPKD patients under metformin treatment and to evaluate any possible effect of metformin on ADPKD progression. In these patients, we have evaluated the modification of kidney function in the last 3 years, compared to a group of non-diabetic ADPKD.

## Methods

We selected our study population from a total available pool of 300 ADPKD patients.

The selected ADPKD diabetic patients (*n* = 7, Metformin Group) met the following inclusion criteria: (a) age ≥ 18 and ≤ 65 years; (b) anamnestic and clinical diagnosis of progressive ADPKD, with a baseline ultrasound kidney length ≥ 16.5 cm [14, 15]; (c) CKD stage 3 by MDRD formula at baseline, 3 years before the last observation (eGFR < 60 ml/min); (d) concomitant type 2 diabetes in continuous treatment with metformin in the last 3 years, with a minimum dosage of 500 mg × 2/day. This dosage was requested because preclinical studies have suggested, by extrapolating experimental data to humans, that a daily dose of ∼1000–1500 mg should activate AMPK in patients [16].

As Control Group, we selected 7 ADPKD patients with at least 3 years of follow-up, matched for sex, age, and basal eGFR (i.e. of the preceding 3 years) and the same anamnestic and clinical evidence of severity and progressive disease at baseline. Informed and written consent for treatment of data was obtained by all the patients.

### Statistical methods

Variables were summarised using descriptive statistics and compared between group using standard statistical techniques (T test, Mann Whitney U Test and Fisher Exact test). In order to compare the longitudinal course of eGFR in the two groups, a linear mixed model with random intercept was used, with time coded continuously. The difference in slopes between the two groups was assessed by adding the interaction term time x group in the model. Results of the LMM were expressed as annual change with the corresponding 95% Confidence Intervals. Statistical significance was set at *p* < 0.05.

## Results

Data were collected at the last follow-up visit and, retrospectively, during the preceding 3 years, at 12-month intervals (T0, baseline, and T1, T2, T3). The diagnosis of ADPKD was made on the basis of family history and of clinical criteria [17]. As shown in Table 1, at baseline (T0) both demographic and clinical data of the 2 groups of patients were comparable, with exception of glucose levels. It is noteworthy, however, that glycated hemoglobin averaged 6.6 ± 0.2% in Metformin-Group at baseline, that four out of 7 patients had no urine protein excretion, although one had a nephrotic proteinuria, and that no patient was affected by vascular diabetic complications, denoting a good metabolic control of the disease. At baseline, patients of control group showed a similar severity of renal disease, and 3 out of 7 had no proteinuria.

**Table 1:** Baseline characteristics of patients

	Metformin (*n* = 7)	Controls (n = 7)	*p* value
Male Gender	3 (43)	3 (43)	1
Age (years)	53.3 ± 7.8	52.9 ± 7.4	0.918
Weight (kg)	99.4 ± 25.6	79.9 ± 11.6	0.091
BMI (kg/m^2^)	34.1 ± 8.8	28.0 ± 3.5	0.118
Blood Pressure (mm Hg)			
Systolic	131.4 ± 9.0	140 ± 15.3	0.225
Diastolic	85.0 ± 5.8	85.0 ± 7.6	1.000
Serum creatinine (mg/dl)	1.51 ± 0.36	1.54 ± 0.40	0.875
eGFR (mL/min per 1·73 m^2^)^a^	48.1 ± 11.1	48.0 ± 15.5	0.994
Haemoglobin (g/dl)	14.5 ± 2.3	13.4 ± 1.3	0.298
Urine proteins (g/24 h)	0 [0; 3990]	131 [0; 350]	0.143
Fasting serum glucose (mmol/L)	118.7 ± 17.6	91.3 ± 9.7	0.012
Blood pressure lowering drugs (n)	1.71 ± 0.8	1.71 ± 1.0	1
ACE- ARBs use	6 (86)	6 (86)	1

Data are expressed as mean ± standard deviation, Median [range] or n (%)

*Abbreviations*: *GFR* glomerular filtration rate, *ACE-I* Corverting enzyme inibitors, *ARBs* Angiotensin receptors blockers

^a^Measured by Estimated by the four-variable equation from Modification of Diet in Renal Disease study

**Fig. 1:**
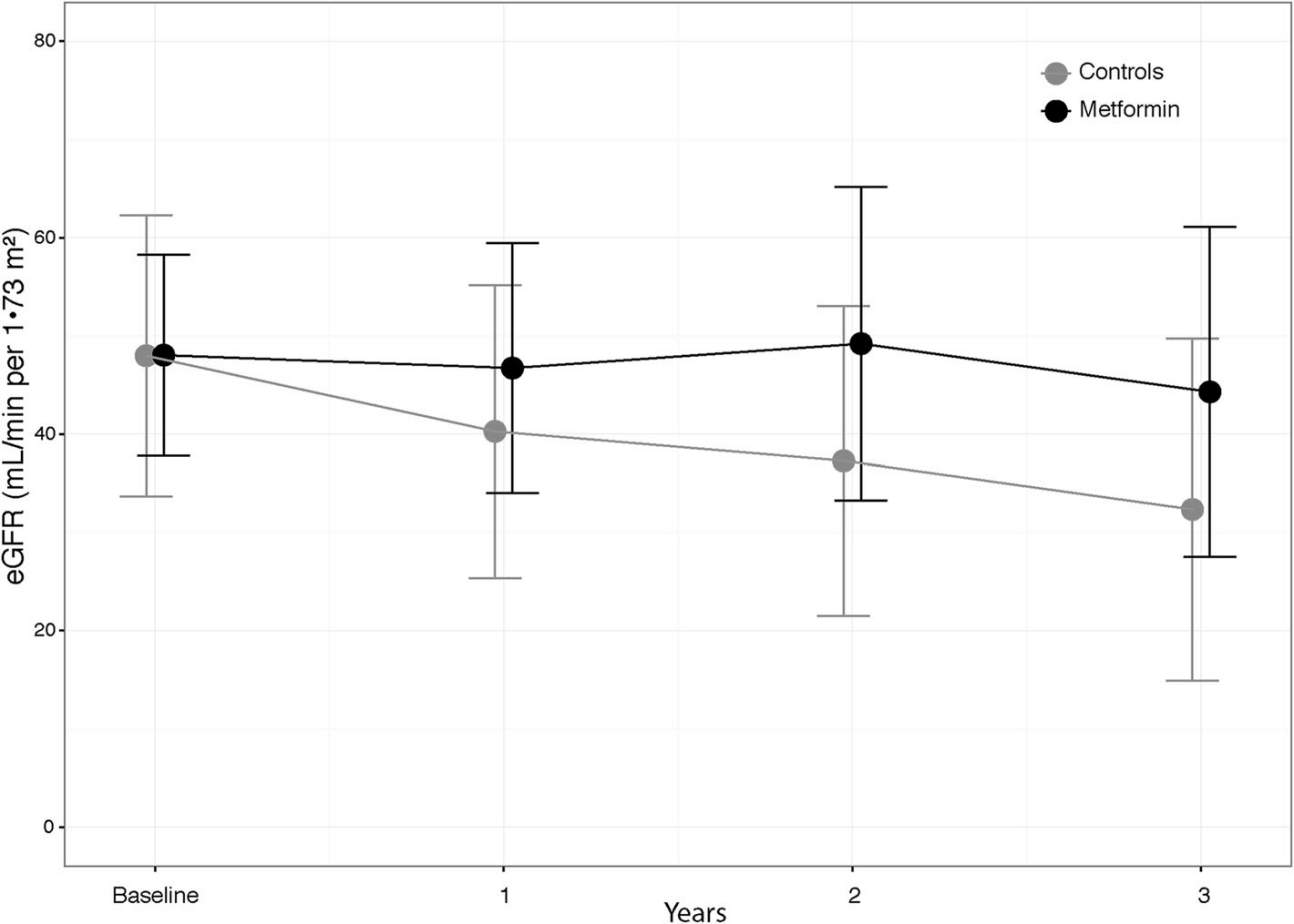
Modification of eGFR during the 3-year follow-up period in Metformin Group (black dots) and Control Group (grey dots). For Group details, see text

The mean dosage of Metformin in these patients was 1833 ± 258 mg/day, and was maintained throughout the study, and treatment length averaged 5.2 ± 1.7 years (range: 3.6–7.2). During the first year of observation (T0 to T1), retrospective data showed that the GFR decreased by 2.5% in the Metformin Group and by 16% in Control patients (Fig. 1). Thereafter, renal function remained quite stable in Metformin Group and further decreased in Control Group, reaching a 50% difference in GFR decline rate between Groups from T1 to T3 (Fig. 1); accordingly, the overall crude loss of GFR (T0-T3), estimated by a linear mixed model, resulted slower in the Metformin than in Control Group (− 0.9; 95% C.I.: -2.7 to 0.9 vs - 5.0; 95% C.I.: -6.8 to − 3.2 mL/min/1.73 m2 per year, *p* = 0.002).

Throughout the 3-year follow-up period, blood pressure remained constant in both Groups, and no difference were detected in main laboratory data within and between the Groups, nor in 24-h urine volume, with exception of serum urea concentration, slightly higher in Control Group at T1 and T2 (data not shown). The value of glycated hemoglobin (6.2 ± 0.3% at the end of the study) remained well controlled during 3-year of follow-up highlighting a satisfactory control of the diabetic disease. Six patients of both groups remained on ACE-inhibitors or ARB treatment during the observation period; the use of antihypertensive drugs and of other therapies was comparable between the Groups and was maintained throughout the follow-up. No patient used additional antidiabetic drugs. Only two hypoglycemic episodes (in a single patient) were reported as drug-related adverse effects in Metformin Group.

## Discussion

To our knowledge, this is the first report that suggests a potential beneficial effect of metformin in delaying the progression of renal dysfunction in ADPKD patients with moderately impaired GFR. The results of this preliminary observation deserve attention for several reasons. First, the rate of GFR decline was extremely reduced in Metformin patients compared to controls, with an annual slope of decrease even lower than those reported in previous controlled trials. Second, GFR remained quite stable throughout the observation period, indicating a sustained effect with time. Third, metformin had an enviable safety profile, with no serious side effect in the setting of ADPKD. Last, GFR was better preserved in metformin treated patients, despite the presence of diabetes as further comorbidity, and of a BMI 22% higher than in controls: it is well known that diabetes and obesity have a negative impact on renal function and proteinuria which, conversely, were not modified throughout the 3-year follow up [18].

Unfortunately, this enthusiastic representation is deeply challenged by a series of limits. First, there is no direct proof that the stability of GFR is really due to metformin action although, beyond the presence of diabetes and the use of metformin, there was no substantial difference between the groups. Second, the progressive nature of ADPKD in Metformin patients was postulated only on anamnestic data (at least one relative starting dialysis before the age of 60) and on ultrasound data (kidney length > 16.5 cm in patients younger than 45). Indeed, our patients were older than 45, but, given their progression, we cannot exclude that this requirement was already present at 45 years of age; on the other hand, it seems really difficult to hypothesize that, considering renal size, our patients had a non-progressive ADPKD disease. Third we evaluated renal function by a calculated GFR like in TEMPO trial [6] and not by iohexol, the golden standard technique [19], like in the ALADIN trial [5], and could not perform repeated MRI or CT to evaluate changes in total kidney volume. The last limit resides in the retrospective nature of the study and in the exiguous number of investigated patients: we cannot exclude a selection bias, although we did enroll in the study all the diabetic patients under metformin responding to our strict inclusion criteria. Nevertheless, despite these shortcomings, our data strongly suggest a sustained beneficial effect of metformin on renal disease progression; the rate of GFR decline during metformin (− 1.3 mL/min/1.73 m^2^ per year), in fact, resulted even lower than that observed in the TEMPO trial (− 2.61 mL/min/1.73 m^2^ per year) [6], or in the ALADIN trial (− 3.81 mL/min/1.73 m^2^ per year) [5]. Moreover, both last trials recruited patients with a better preserved renal function (basal eGFR> 60 ml/min) and without diabetes.

The aim of this brief report is to suggest Nephrologists to consider the use of metformin in all diabetic patients with ADPKD and a preserved GFR, and to offer their eligible patients to the recruiting trials aimed to evaluate the feasibility of metformin to slow ADPKD progression. We also invite all Nephrologists to examine the records of metformin-treated ADPKD patients of their own databases, as we did, to evaluate the rate of progression of renal disease and to publicize their data. We need urgent answers to our queries to transform the Myth that we have described into Facts!

We must not forget that metformin is currently administered to million patients for its good metabolic and safety profile. Beyond some gastrointestinal symptoms, its most serious side effect, lactic acidosis, is observed only when GFR is below 30 ml/min [20], far below the cut-off value accepted for a patient to enter the ongoing trials (eGFR> 60 ml/min). This profile will be hopefully maintained also in non-diabetic patients and the fearsome risk of hypoglycemia should remain negligible: clinical trials using metformin in diabetes prevention, in fact, have shown no case of hypoglycemia occurring as serious adverse effect during nearly 18.000 subjects/year of follow-up [21], nor hypoglycemia is described in obese non-diabetic children assuming the drug [22], nor in normal subjects after an acute load of metformin [23]. Such tolerability is a crucial point in a long lasting treatment. A comparison with tolvaptan adverse effects, as described in TEMPO study [6], seems superfluous.

Last, we must also remember that, beyond this potentially optimal risk–benefit profile, the cost of this old drug is dramatically lower than that of all the previously used drugs: billion dollars could be saved in the long-term!

## Conclusions

In conclusion, this preliminary observation is suggestive of a beneficial effect of metformin on the progression of ADPKD. Whether this drug will represent an option for long-term treatment of ADKPD, however, must be confirmed by ongoing trials, for which we need a large enrollment of patients.

## Abbreviations

ADPKD: Autosomal dominant polycystic kidney disease
AMPK: AMP-activated protein kinase
CFTR: Cystic fibrosis trans-membrane conductance regulator
CKD: Chronic kidney disease
